# Does local matter? Evaluating susceptibility variations between hospital-wide and hematology-oncology unit antibiograms

**DOI:** 10.1017/ash.2022.36

**Published:** 2022-06-21

**Authors:** Rachel Bartash, Erika Orner, Kelsie Cowman, Wendy Szymczak, Priya Nori, Noah Kornblum, Margaret McCort

**Affiliations:** 1 Division of Infectious Diseases, Department of Medicine, Montefiore Medical Center, Albert Einstein College of Medicine, Bronx, New York; 2 Department of Pathology, Montefiore Medical Center, Albert Einstein College of Medicine, Bronx, New York; 3 Department Oncology, Montefiore Medical Center, Albert Einstein College of Medicine, Bronx, New York

## Abstract

Antibiograms are important for guiding empiric antibiotics for febrile neutropenia. However, hospital-wide antibiograms may not capture complexities of patients with hematologic malignancies. We created a hematology-oncology unit-specific antibiogram and found higher resistance among *Escherichia coli*, *Klebsiella pneumonia*, and *Enterococcus* isolates compared to hospital-wide data.

Antibiograms are important stewardship tools that reflect evolving trends in antibiotic resistance and pathogen prevalence.^1^ However, most antibiograms are hospital-wide and microbiologic data may vary between hospital units and patient subgroups, particularly those with higher antibiotic exposure, including intensive care unit (ICU) and transplant patients.^1,2^

Individuals with hematologic malignancies are unique because immunosuppression, chemotherapy regimens, and stem cell transplantation often necessitate antibiotic prophylaxis. Additionally, these patients are exposed to extended courses of broad-spectrum antibiotics during prolonged hospital admissions.^3^ Extensive antibiotic exposures can increase the rate of resistant infections among these patients.^3^ Kokkayil et al^4^ found that 47% of isolated gram-negative organisms in patients with neutropenic fever were categorized as extensively drug resistant. Conversely, another study found widespread fluoroquinolone susceptibility in this population,^5^ suggesting variable regional resistance patterns and further reinforcing the need for local antibiograms.

Effective empiric antibiotics are critical for febrile neutropenia management, and antibiograms can play an important role in guiding therapy. We hypothesized that a unit-specific antibiogram for our hematology–oncology unit would reveal a higher incidence of multidrug resistance and reduced susceptibility to frequently prescribed antibiotics compared to hospital-wide data.

## Methods

We reviewed all positive cultures with antimicrobial susceptibilities collected on our 32-bed hematology–oncology unit from July 2016 through June 2020. Clinical cultures from all sites were included (ie, urine, blood, respiratory, wound). Only the first isolate for each patient was included to avoid duplicates. Isolates from patients with hematologic malignancies collected outside the unit were not included. Antimicrobial susceptibility testing was performed using the Phoenix system (Becton-Dickinson, Franklin Lakes, NJ) and break points established by the Clinical and Laboratory Standards Institute (CLSI).^6^ Based on CLSI recommendations, only organisms with ≥30 isolates were included in antibiogram analysis. Data collection was extended to 4 years due to the low number of positive cultures.

Bacterial isolates and antibiotic susceptibilities for all cultures performed in our 727-bed hospital are compiled and reviewed annually by the microbiology laboratory and used to synthesize the hospital-wide antibiogram. These data include all clinical cultures, including those collected from hematology–oncology patients on and off the unit. Hospital-wide antibiogram data from July 2016 through June 2020 served as a comparator.

The number of isolates reported on both antibiograms reflects the highest number of isolates tested for any individual antibiotic. Susceptibilities were compared using the χ^2^ or the Fisher exact test, as appropriate. The number of patients undergoing stem-cell transplantation (SCT) and unit antibiotic utilization rate from July 2016 through June 2020 were also evaluated.

## Results

An average of 94 autologous and 21 allogeneic SCTs occurred yearly on this unit. A high utilization of prophylactic and therapeutic antibiotic use was observed (493 days of therapy of any antibiotic per 1,000 days present).

Overall, 522 bacterial isolates were obtained on the hematology–oncology unit, representing 56 organisms. Among them, 7 organisms met CLSI criteria: *Escherichia coli* (n = 102), *Klebsiella pneumoniae* (n = 38), *Pseudomonas aeruginosa* (n = 33), *Staphylococcus aureus* (n = 61), *Staphylococcus epidermidis* (n = 45), *Enterococcus faecalis* (n = 36), and *E. faecium* (n = 53). Due to difficulties interpreting clinical significance, *Staphylococcus epidermidis* was excluded. The hospital-wide antibiogram included 24,752 isolates, representing 157 organisms.

Compared to the hospital-wide antibiogram for most used antibiotics, higher rates of resistance occurred among *Escherichia coli* and *Klebsiella pneumoniae* in the unit-specific antibiogram. The greatest disparity was noted among fluoroquinolones (Table 1). There were no differences in susceptibilities among *Pseudomonas aeruginosa* (Table 1). However, *E. faecalis* and *E. faecium* isolates in the unit-specific antibiogram were more likely to be vancomycin resistant (33.3% vs 10.2%, *P* = .043; 7.6% vs 23%, *P* = .008). We did not detect a difference in the rate of methicillin-resistant *Staphylococcus aureus* (MRSA). Additional results are shown in Table 2.

**Table 1.** Percentage of Gram-Negative Isolates Susceptible to Specific Antibiotics Based on Unit-Based Antibiogram Versus Hospital-wide Antibiogram^a^

Antimicrobial Agent	*Escherichia coli*			*Klebsiella pneumoniae*			*Pseudomonas aeruginosa*		
	Unit (n = 102)	Hospital (n = 6,040)	*P* Value	Unit (n = 38)	Hospital (n = 2,611)	*P* Value	Unit (n = 33)	Hospital (n = 1,731)	*P* Value
Aztreonam	67.65	81.73	**.001**	60.53	77.78	**.017**	69.70	69.14	>.99
Ceftriaxone	67.33	81.45	**.001**	60.53	77.59	**.018**	0	0	…
Cefepime	71.29	82.97	**.005**	60.53	78.28	**.0159**	90.63	86.04	.610
Ciprofloxacin	44.55	63.00	**<.0001**	65.79	80.62	**.036**	87.88	77.79	.205
Levofloxacin	45.10	65.03	**<.0001**	71.05	87.45	**.0062**	87.50	76.98	.203
Meropenem	98.04	99.60	.069	97.37	95.67	>.99	81.82	83.57	.812
Piperacillin-tazobactam	65.69	77.80	**.006**	50.00	73.92	**.002**	78.79	78.34	>.99
Tobramycin	74.51	83.23	**.045**	65.79	83.97	**.006**	100	96.24	.63
Amikacin	100	99.69	>.99	97.37	97.85	.565	100	99.02	>.99

a The reported number of isolates (n) reflects the total number of organisms tested. Bold values indicate statistical significance. The n may not reflect slight variations for some antibiotics due to occasional antibiotic well termination on susceptibility testing panels. Antibiotic well termination occurs when there are inconsistencies in the organism growth pattern across the antimicrobial concentration range causing testing for the antibiotic to be terminated.

**Table 2.** Percentage of Gram-Positive Isolates Susceptible to Specific Antibiotics Based on Unit-Based Antibiogram Versus Hospital-wide Antibiogram^a^

Antimicrobial Agent	*Staphylococcus aureus*			*Enterococcus faecalis*				*Enterococcus faecium*			
	Unit (n = 61)	Hospital (n = 3,289)	*P* Value	Antimicrobial Agent	Unit (n = 36)	Hospital (n = 1,539)	*P* Value	Antimicrobial Agent	Unit (n = 53)	Hospital (n = 545)	*P* Value
Oxacillin	54.10	58.86	.6897	Ampicillin	94.44	98.50	.110	Ampicillin	1.89	9.54	.073
Vancomycin	100	99.97	>.99	Vancomycin	66.67	89.85	**.043**	Vancomycin	7.55	23.02	**.008**
Trimethoprim-sulfamethoxazole	95.08	93.81	.98	Daptomycin	100	97.72	1.000	Daptomycin	88.68	93.94	.144
Tetracycline	95.08	88.99	.149	Linezolid	100	98.76	1.000	Linezolid	96.23	93.58	.763
Linezolid	100	99.94	.99								
Clindamycin	68.85	71.44	.668								

a The reported number of isolates (n) reflects the total number of organisms tested. Bold values indicate statistical significance. The n may not reflect slight variations for some antibiotics due to occasional antibiotic well termination on susceptibility testing panels. Antibiotic well termination occurs when there are inconsistencies in the organism growth pattern across the antimicrobial concentration range causing testing for the antibiotic to be terminated.

## Discussion

We found higher resistance among *Klebsiella pneumoniae*, *Escherichia coli*, and *Enterococcus* isolates obtained from a hematology–oncology unit compared to isolates from the hospital-wide antibiogram. We attribute this difference to risk factors for development of MDRO infections, including prolonged neutropenia, extensive hospital stays, and reliance on prophylactic antibiotics. Our results align with those of Rosa et al,^2^ who found that antibiograms for solid-organ transplant units had significantly higher rates of resistance compared to hospital-wide data.

Higher rates of resistance among certain gram-negative organisms suggest a need for heightened stewardship. Even though antibiotics are critical medications, judicious use is necessary to prevent resistance, which, if unchecked, makes classes of antibiotics ineffective for patients who may need them the most. Data suggest that shorter duration of antibiotics for neutropenic fever is not inferior to more prolonged courses.^8^ Stewardship targeting duration of therapy may help prevent or slow development of antibiotic resistance without compromising clinical outcomes.

In addition to increased gram-negative resistance, vancomycin resistance was significantly higher in our unit-specific antibiogram among both *E. faecalis* and *E. faecium*. We suspect that this is at least in part due to frequent use of empiric vancomycin in patients with febrile neutropenia, which is often administered outside recommended guidelines.^9^ Because vancomycin is often started for MRSA coverage, it is important to note that rates of MRSA were similar on both antibiograms. Previous studies have also found low rates of MRSA colonization among hematology–oncology patients.^9^ One reason for these low rates of colonization may be due to the limited anti-staphylococcal activity of fluoroquinolones, the agents most used for neutropenic prophylaxis. Therefore, although widespread fluoroquinolone use may contribute to increased gram-negative resistance, the same pressure may not apply to *Staphylococcus* isolates. Given high rates of vancomycin-resistant *Enterococcus* and >50% methicillin-sensitivity among *S. aureus* isolates, ongoing stewardship efforts targeting the use of vancomycin on hematology–oncology units is needed.

Interestingly, differences in resistance patterns were not observed for *Pseudomonas aeruginosa*, the third most commonly isolated gram-negative organism. This finding, similar to that of Kern et al,^10^ may be due to the nature of acquired resistance mutations for various organisms. Enterobacteriaceae, including *K. pneumoniae* and *E. coli*, typically utilize β-lactamases or plasmid-borne cephalosporinases to confer resistance to β-lactam antibiotics, or they develop chromosomal mutations conferring resistance to fluoroquinolones.^7^ Conversely, *Pseudomonas* preferentially utilizes specific efflux pumps as a resistance mechanism. Differences in acquisition and mechanism of antibiotic resistance may contribute to the lack of difference noted among our *P. aeruginosa* isolates, though this may also be explained by higher baseline rates of fluoroquinolone resistance on our hospital-wide antibiogram.

This study had several limitations. The number of unit-specific antibiogram isolates included was small. Based on CSLI recommendations, we were only able to evaluate 6 organisms and additional patterns of resistance might not have been recognized due the low frequencies of other isolates. Additionally, hematologic malignancy patients can be admitted to other hospital units, and their clinical isolates are included in the hospital-wide antibiogram only. Furthermore, the hospital-wide antibiogram included cultures obtained on the hematology–oncology unit, so these data are present in both antibiograms. However, these represent a small proportion of hospital-wide antibiogram isolates, and given the increased resistance seen on the unit-specific antibiogram, inclusion in the hospital-wide antibiogram should have decreased the differences noted between antibiograms.

In summary, hematology–oncology unit-specific antibiograms are important clinical decision tools for empiric treatment of neutropenic fever because increased drug resistance may be seen on these units. Local resistance patterns can help drive antibiotic stewardship efforts in this high-risk population.
